# Nitric oxide down-regulates voltage-gated Na^+^ channel in cardiomyocytes possibly through S-nitrosylation-mediated signaling

**DOI:** 10.1038/s41598-021-90840-0

**Published:** 2021-05-28

**Authors:** Pu Wang, Mengyan Wei, Xiufang Zhu, Yangong Liu, Kenshi Yoshimura, Mingqi Zheng, Gang Liu, Shinichiro Kume, Masaki Morishima, Tatsuki Kurokawa, Katsushige Ono

**Affiliations:** 1 Department of Cardiology, The First Hospital of Hebei Medical University, 89 Donggang Road, Shijiazhuang, 050031 Hebei Province People’s Republic of China; 2 Department of Food Science and Nutrition, Faculty of Agriculture, Kindai University, Nara, Japan; 3 Department of Pathophysiology, Oita University School of Medicine, Yufu, Oita 879-5593 Japan

**Keywords:** Physiology, Cardiology

## Abstract

Nitric oxide (NO) is produced from endothelial cells and cardiomyocytes composing the myocardium and benefits cardiac function through both vascular-dependent and—independent effects. This study was purposed to investigate the possible adverse effect of NO focusing on the voltage-gated Na^+^ channel in cardiomyocytes. We carried out patch-clamp experiments on rat neonatal cardiomyocytes demonstrating that NOC-18, an NO donor, significantly reduced Na^+^ channel current in a dose-dependent manner by a long-term application for 24 h, accompanied by a reduction of Nav1.5-mRNA and the protein, and an increase of a transcription factor forkhead box protein O1 (FOXO1) in the nucleus. The effect of NOC-18 on the Na^+^ channel was blocked by an inhibitor of thiol oxidation *N*-ethylmaleimide, a disulfide reducing agent disulfide 1,4-Dithioerythritol, or a FOXO1 activator paclitaxel, suggesting that NO is a negative regulator of the voltage-gated Na^+^ channel through thiols in regulatory protein(s) for the channel transcription.

## Introduction

Nitric oxide (NO) is a small gaseous molecule implicated in multiple signal transduction pathways, and is a critical factor in preventing the pathogenesis and progression of cardiovascular diseases heart^1^. Recent studies revealed that NO plays an important role in the development of cardiovascular disease^2,3^. NO is also recognized as a therapeutic drug classified as a vasodilator, and its activity is mediated through stimulation of soluble guanylate cyclase (cGC), which results in formation of the second messenger cyclic guanosine monophosphatase (cGMP) and increased activity of protein kinase G (PKG)^4,5^. Moreover, it is recently clarified that NO is also capable of signaling independent of cGMP, namely via post-translational modulation of protein thiol groups. This redox-sensitive modification is known as protein S-nitrosylation^6–8^. Although NO-dependent modulation of cardiac function is intensively studied in the past decades in the diseased and failing heart, several aspects of NO signaling in the myocardium remain poorly understood. Some apparently contrasting findings may have arisen from the use of non-isoform-specific inhibitors of NO synthase as compared to the use of experimental models genetically deficient or overexpressing the NO synthase^9–11^. The challenge is now to highlight these emerging findings on the critical role of NO in cardiac physiology/pathophysiology.

In cardiovascular system many ion channels regulate transmembrane currents that make much contribution to excitation in cardiomyocytes^12–14^. Among them the voltage-gated Na^+^ channel current (*I*_Na_) plays a critical and significant role for the fast upstroke of the action potentials and is essential for proper conduction of the electrical impulse. Voltage-gated sodium channels are composed of a pore-forming α subunit and auxiliary β subunits. SCN5A gene encodes Na_V_1.5 protein, the α-subunit of the major cardiac voltage-gated sodium channel. Although several studies have revealed an acute action of NO to induce persistent Na^+^ current in cardiomyocytes^15,16^, long-term effects of NO on the channel gating and expression of the channel protein are poorly understood. The present study provides novel information that NO is a negative regulator of the voltage-gated Na^+^ channel possibly through S**-**nitrosylation pathway in regulatory proteins including forkhead box protein O1 (FOXO1) for the channel transcription.

## Results

### Long-term effects of an NO donor NOC-18 on I_Na_

**Figure 1:**
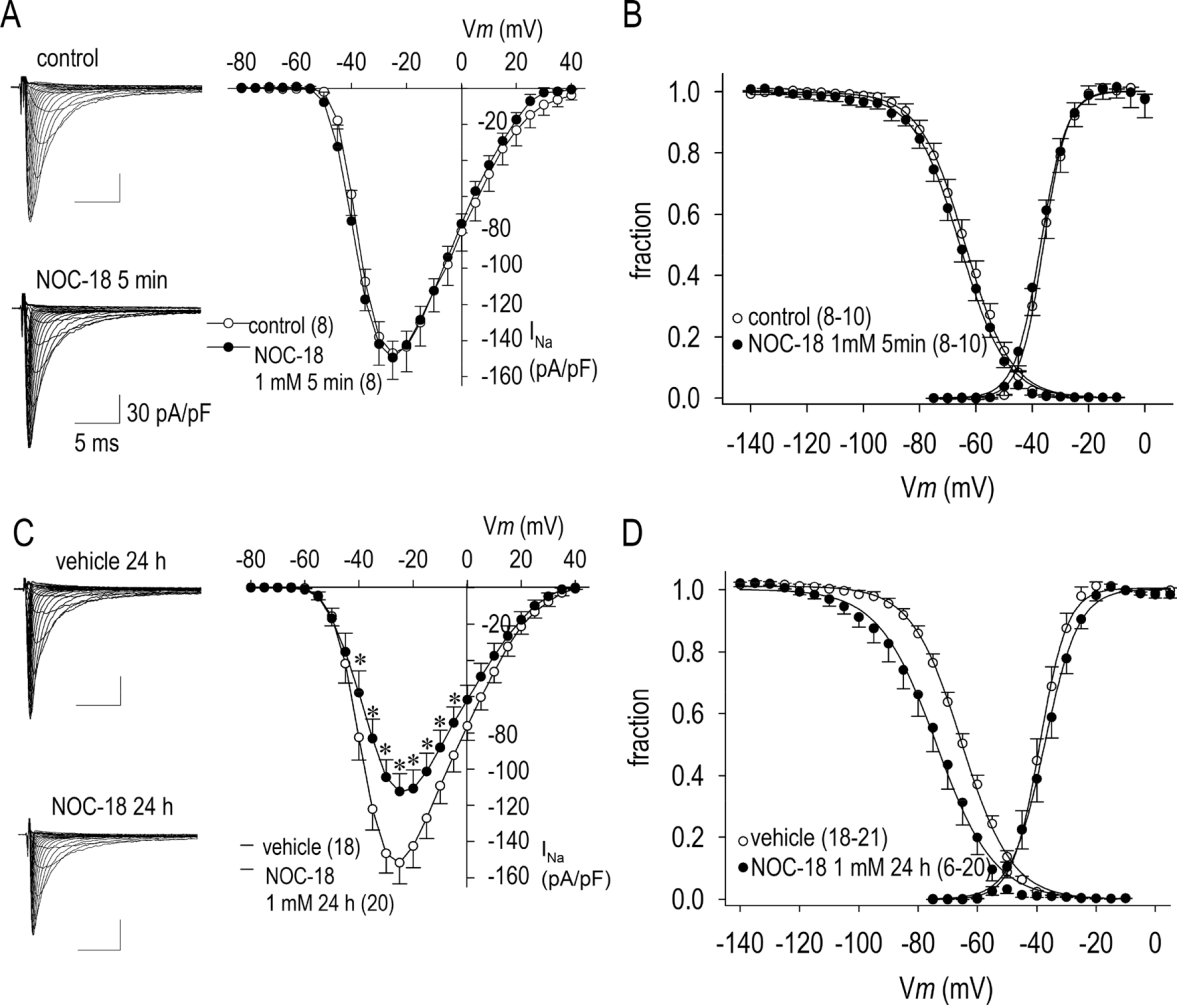
Short- and long-term effects of NOC-18 on Na^+^ channel current (I_Na_) in neonatal rat cardiomyocytes. (**A**) Representative I_Na_ traces in the control condition and during the acute (5 min) application of 1 mM NOC-18 given to the same patch are shown. I_Na_ was recorded by a depolarization pulse of 50 ms duration, ranging from − 80 to 40 mV in 5 mV steps, applied from holding potentials of − 140 mV. Current (I)–voltage (V) relationship before (control) and after application of 1 mM NOC-18 (5 min) demonstrates the maximum inward current of the control (− 151.2 ± 14.9 pA/pF) and with NOC-18 (− 156.2 ± 9.5 pA/pF). (**B**) The steady-state inactivation and activation (conductance) curves of the control and during application of NOC-18 (5 min); Mid-points of the voltage relation for the activation (*V*_a,1/2_) was − 36.0 ± 0.2 mV in the control and − 37.1 ± 0.2 mV during application of NOC-18; Midpoint of the voltage relation for the inactivation (*V*_i,1/2_) was − 63.7 ± 0.2 mV in the control and − 65.6 ± 0.2 mV during application of NOC-18. (**C**) Representative I_Na_ traces with vehicle or NOC-18 treated for 24 h. I–V relationship demonstrates the maximum inward current of vehicle (− 160.7 ± 11.6 pA/pF, n = 18) and with NOC-18 (− 122.2 ± 10.9 pA/pF). (**D**) The steady-state inactivation and activation (conductance) curves of vehicle (24 h) and NOC-18 (24 h); V_a,1/2_ was − 39.0 ± 0.2 mV in vehicle and − 37.4 ± 0.2 mV in NOC-18 (24 h); V_i,1/2_ was − 65.3 ± 0.2 mV in vehicle and − 73.8 ± 0.4 mV in NOC-18 (24 h). Values represent the mean ± SE. Numbers of cell are shown in parentheses. *p < 0.05 vs. vehicle.

In conventional whole-cell patch-clamp experiments using rat neonatal cardiomyocytes, *I*_Na_ was recorded. Figure 1A shows representative *I*_Na_ families in the control condition and during the acute action of an NO donor, 2,2′-(hydroxynitrosohydrazino)-bis-ethanamine (NOC-18), for 5 min. Group data indicate that NOC-18 has substantially no acute effect (< 5 min) on I_Na_ as indicated by the current (I)-voltage (V) relationship (Fig. 1A), and the activation (conductance) and the steady-state inactivation curves (Fig. 1B). On the other hand, long-term treatment of cardiomyocytes with 1 mM NOC-18 for 24 h reduced *I*_Na_ by 26% when assessed by the maximum inward current (Fig. 1C). To determine the effect of NOC-18 on the activation and steady-state inactivation kinetics of I_Na_, the fractional *I*_Na_ and fractional Na^+^ channel conductance were compared in cardiomyocytes with or without NOC-18 treatment. Treatment of cardiomyocytes with 1 mM NOC-18 for 24 h had no significant effect on the voltage dependency of the activation curve, which is consistent with the result in Fig. 1C. However, the steady-state inactivation curve was significantly shifted in the direction of hyperpolarization by 8.5 mV by a long-term treatment with 1 mM NOC-18 (Fig. 1D).

### Dose–response effect of NO donors on I_Na_ suppression

**Figure 2:**
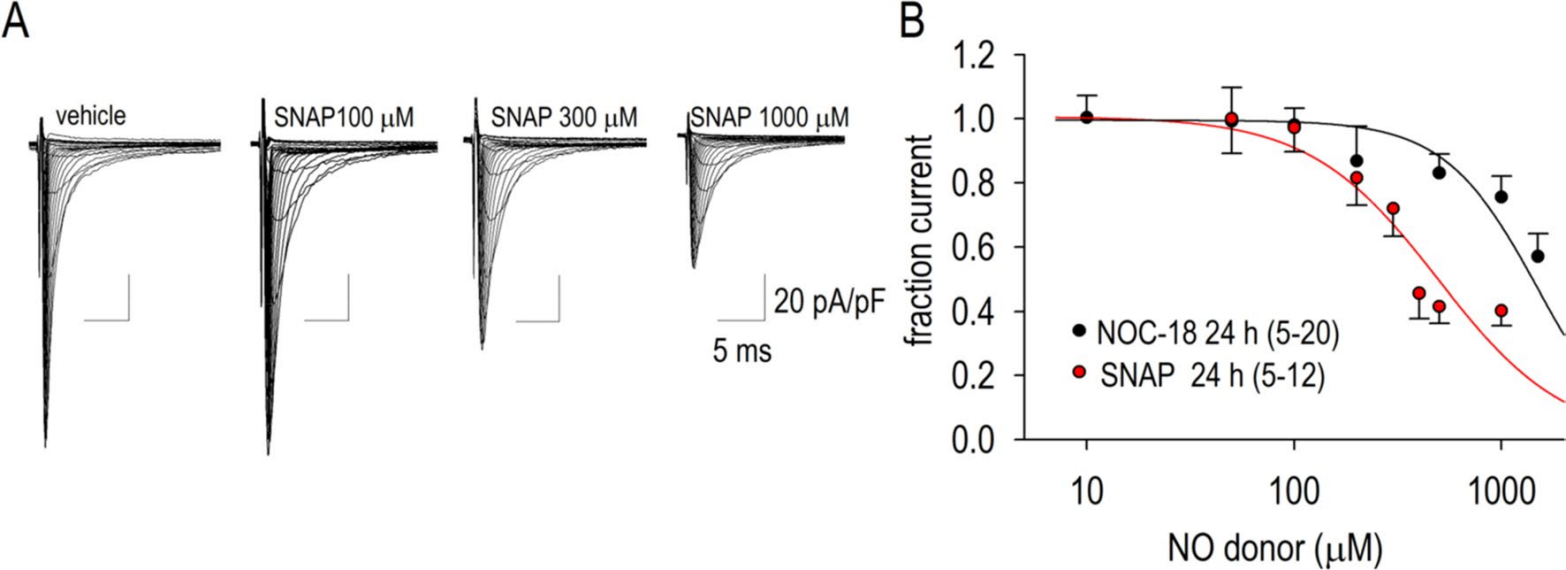
Concentration-dependent long-term (24 h) effects of NOC-18 and S-Nitroso-N-acetyl-DL-penicillamine (SNAP) on I_Na_. (**A**) Representative I_Na_ traces with vehicle, 100 μM SNAP, 300 μM SNAP and 1000 μM SNAP applied for 24 h. (**B**) Dose–response relationships of the effects of NO donors, NOC-18 and SNAP, on the fractional I_Na_. The potency, e.g. half-maximum inhibitory concentration (IC_50_), was estimated as 491 μM and 1505 μM for SNAP and NOC-18, respectively. Numbers of cell are shown in parentheses.

NOC-18 is a diazeniumdiolate analogue with an action of releasing NO^17^. In order to further confirm the action of NO to suppress I_Na_, we have attempted to observe the changes of I_Na_ with a different NO donor without a chemical structure of diazeniumdiolate. S-nitroso-n-acetyl penicillamine, SNAP, is an NO donor with a chemical structure of S–nitrosothiol. Although an acute application of SNAP (300 μM) for 5 min was without effect on I_Na_, treatment of cardiomyocytes with SNAP (300–1000 μM) for 24 h decreased I_Na_ in a dose-dependent manner (Fig. 2A). The half maximal inhibitory concentration (IC_50_) of NOC-18 and SNAP to reduce I_Na_ was 1410 μM and 502 μM, respectively (Fig. 2B).

### Effects of NO scavenger

**Figure 3:**
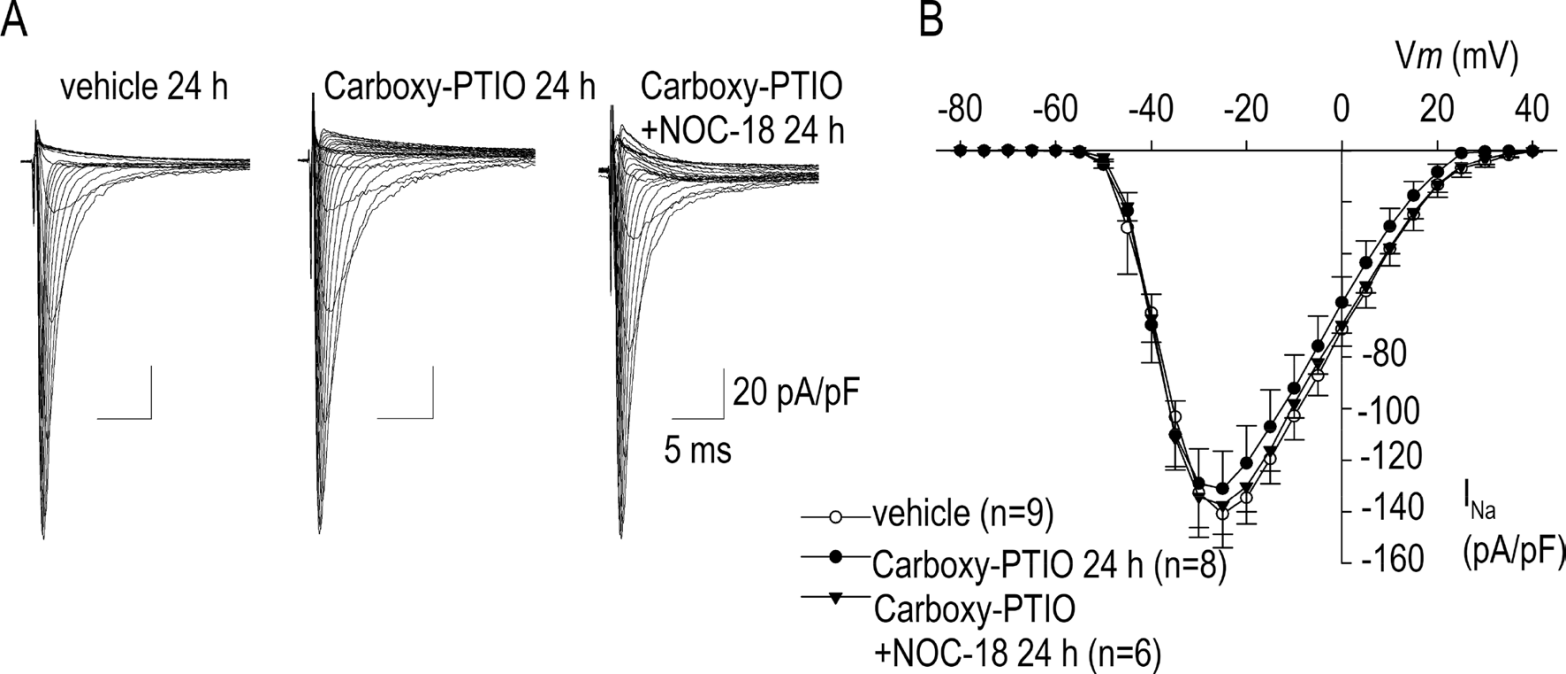
Effects of an NO scavenger carboxy-PTIO (20 μM) on the action of NOC-18 for I_Na_ modulation. (**A**) Representative I_Na_ traces in the control condition (vehicle), 20 μM carboxy-PTIO, and 20 μM carboxy-PTIO plus 1 mM NOC-18 applied for 24 h. (**B**) I–-V relationship of I_Na_ with vehicle, 20 μM carboxy-PTIO, and 20 μM carboxy-PTIO plus 1 mM NOC-18, demonstrating no statistical difference among three groups. Numbers of cell are shown in parentheses. Values represent the mean ± SE.

There is considerable evidence indicating that NO metabolism involves a family of NO-treated molecules in addition to nitrate and nitrate ions. Carboxy-PTIO, carboxy-2-phenyl-4,4,5,5-tetramethylimidazoline-1-oxyl-3-oxide potassium, is a potent NO inhibitor that can make a quick reaction with NO to produce NO_2_^18^. Application of carboxy-PTIO to cardiomyocyte without NOC-18 displayed no appreciable effects on I_Na_. Importantly, NOC-18 (1 mM) was without effect on I_Na_ when cardiomyocytes were co-treated with carboxy-PTIO for the same duration of 24 h (Fig. 3A,B). These results suggest that NOC-18 exerts its effect on I_Na_ mostly through action of NO but not through other metabolites or reactive nitrogen intermediates.

**Figure 4:**
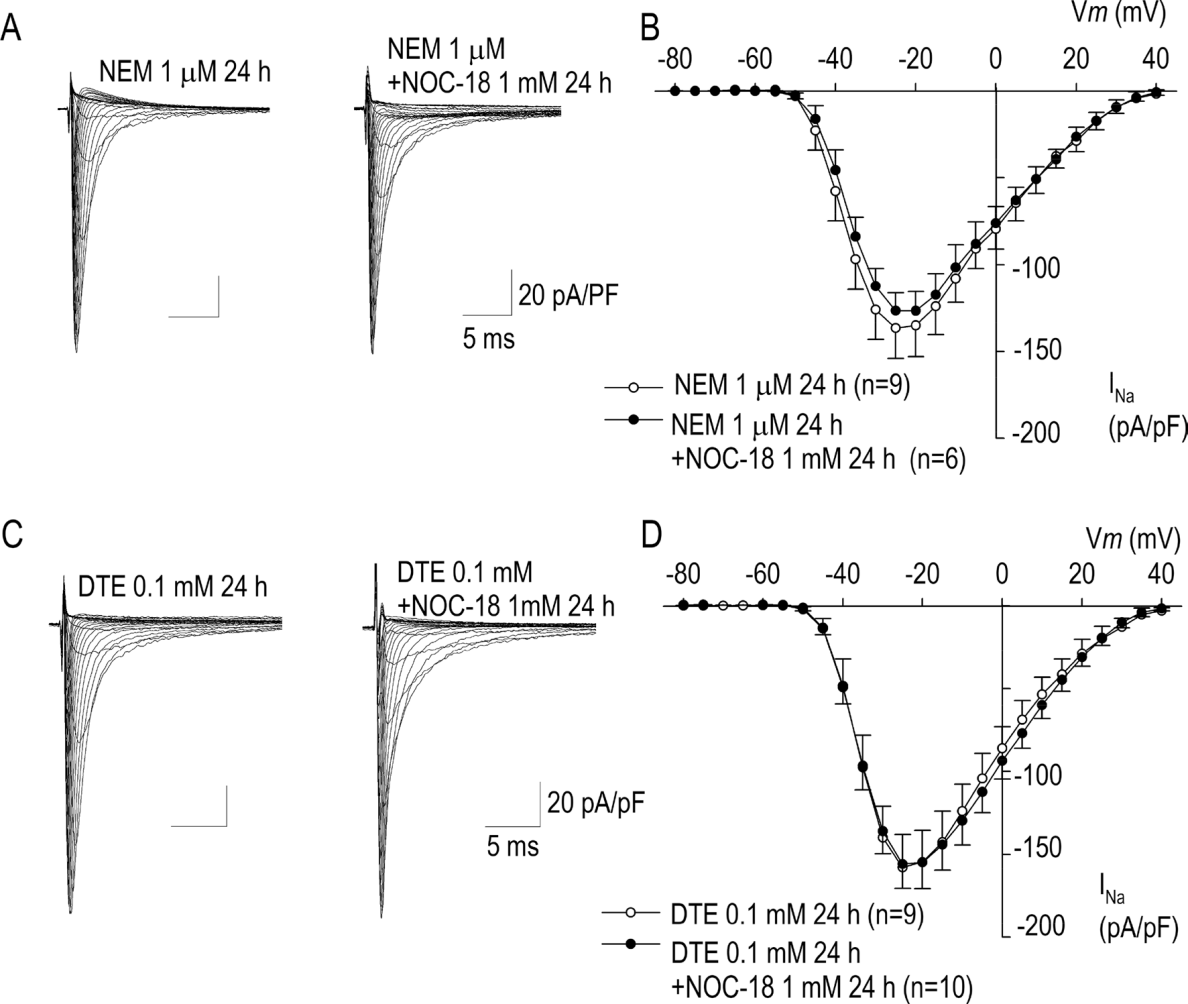
Effects of a sulfhydryl-reducing agent *N*-ethylmaleimide (NEM) and a thiol-reducing agent dithiothreital (DTE) on NOC-18 for I_Na_ modulation. (**A**,**B**) Representative I_Na_ traces with 1 mM NOC-18 applied for 24 h in the absence or presence of NEM, and their I–V relationships of I_Na_. (**C**,**D**) Representative I_Na_ traces with 1 mM NOC-18 applied for 24 h in the absence or presence of DTE, and their I–V relationships of I_Na_. In the presence of NEM or DTE, NOC-18 was unable to reduce I_Na_. Numbers of cell are shown in parentheses. Values represent the mean ± SE.

We evaluated protein thiols as an effector target reflecting NOC-18-dependent regulation of I_Na_ in cardiomyocytes. N-ethylmaleimide (NEM) is a compound that forms stable, covalent thioether bonds with sulfhydryls, enabling them to be permanently blocked to prevent disulfide bond formation^19^; a potent inhibitor of S-nitrosylation as it irreversibly reacts with and binds to sulfhydryl groups thereby preventing NO from engaging in an S-nitrosylation reaction. In the presence of NEM, NOC-18 was unable to reduce I_Na_ when applied for 24 h (Fig. 4A,B). 1,4-Dithioerythritol (DTE) is a strong reducing agent for the quantitative reduction of disulfide groups and cleavage of disulphide bridges in proteins^20^. Likewise, NOC-18 was unable to reduce I_Na_ when applied for 24 h in the presence of DTE (Fig. 4C,D). These results strongly indicate that NO is a negative regulator of cardiac voltage-gated Na channel through thiols in protein(s) possibly responsible for the Na^+^ channel transcription, trafficking or maturation.

### Protein kinase G and S-nitrosylation of thiols

**Figure 5:**
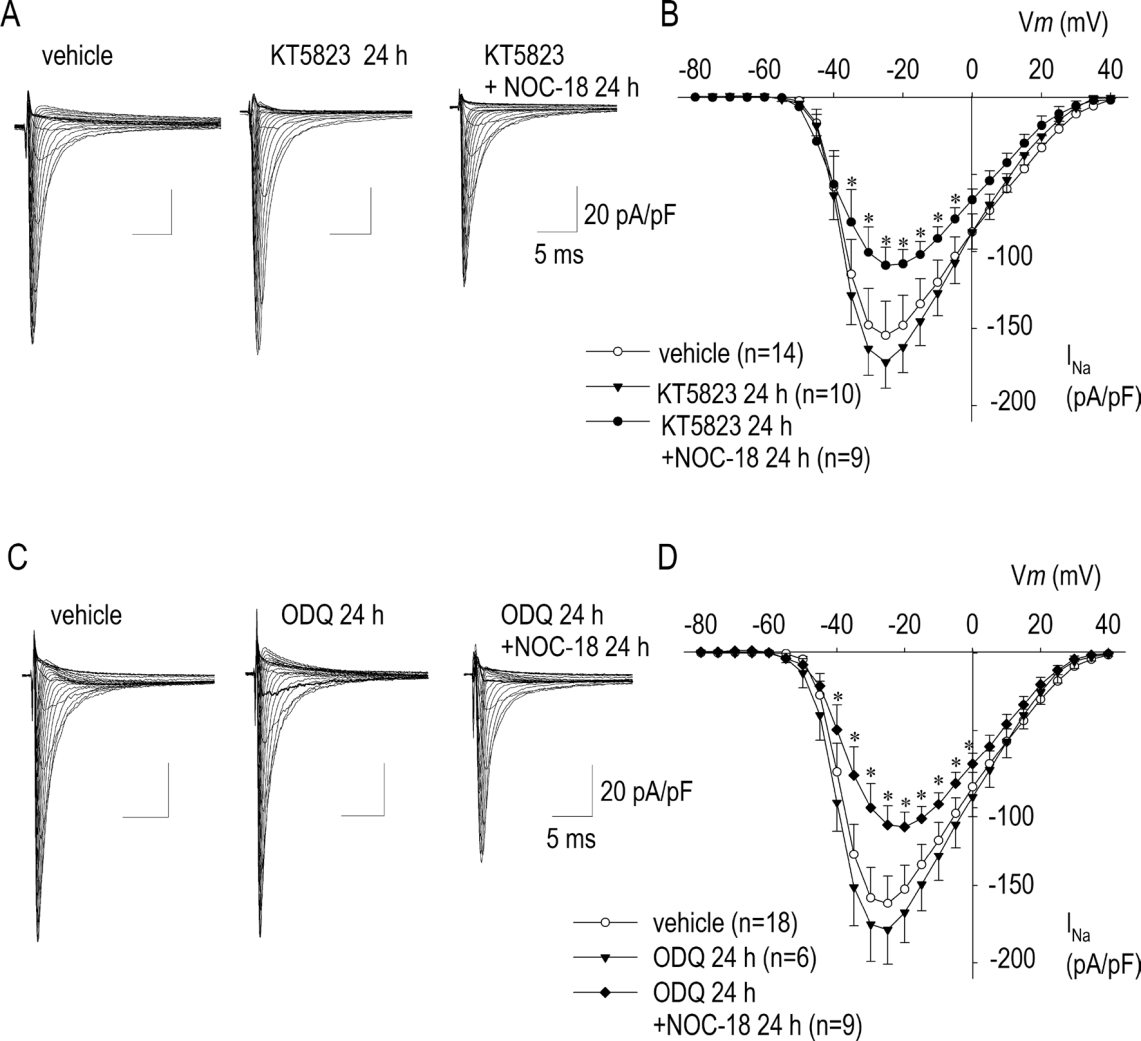
Effects of a PKG inhibitor KT5823 and a guanylate cyclase inhibitor ODQ on NOC-18 for I_Na_ modulation. (**A**,**B**) Representative I_Na_ traces with 1 mM NOC-18 applied for 24 h in the absence or presence of 500 nM, and their I–V relationships of I_Na_. In the presence of KT5823, NOC-18 reduce the maximum inward current of I_Na_ by 31%, indicating that I_Na_ inhibiting effects of NOC-18 was independently of the action through PKG. (**C**,**D**) Representative I_Na_ traces with 1 mM NOC-18 applied for 24 h in the absence or presence of 10 μM ODQ, and their I–V relationships of I_Na_. In the presence of ODQ, NOC-18 reduced the maximum inward current of I_Na_ by 35%, indicating that I_Na_ inhibiting effects of NOC-18 was independently of the action through GC activity. Numbers of cell are shown in parentheses. Values represent the mean ± SE. *p < 0.05 vs. KT5823 + NOC-18 or vs. ODQ + NOC-18.

**Figure 6:**
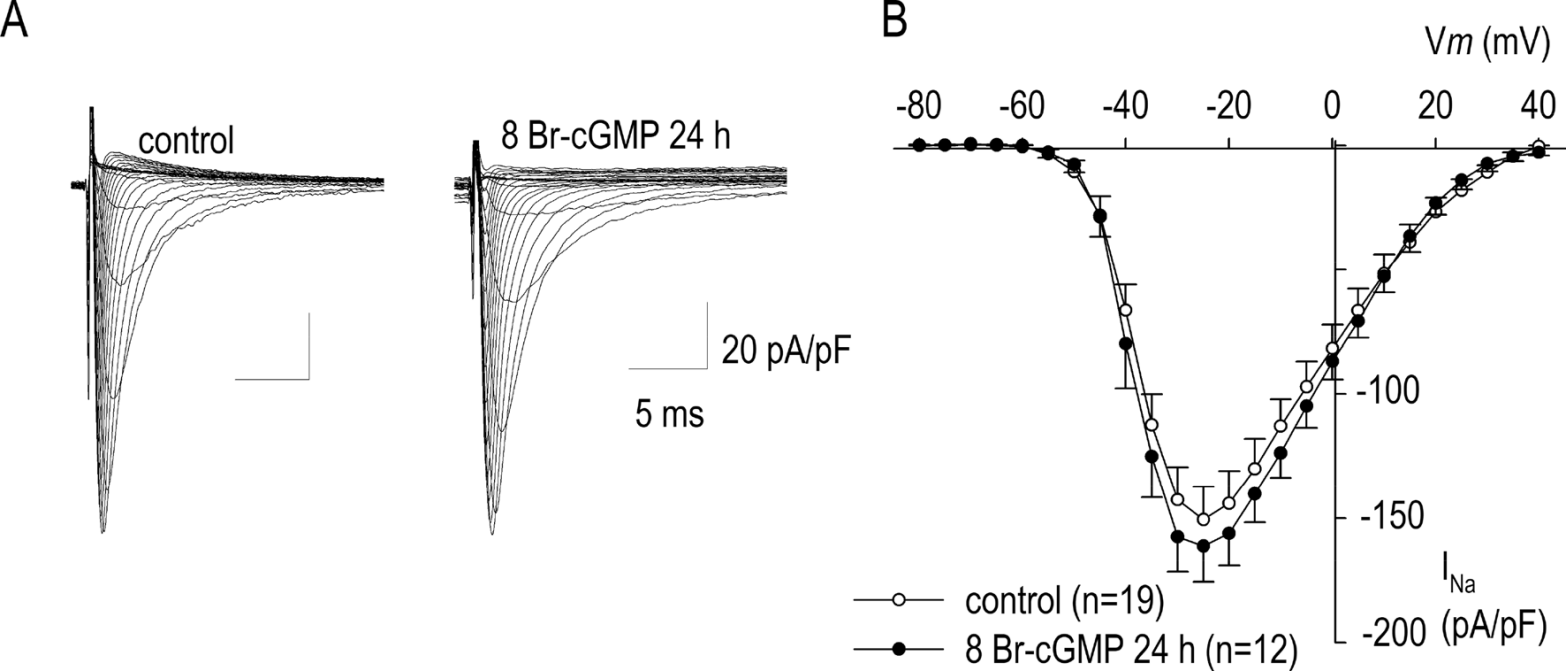
Effects of a membrane-permeable analog of cGMP (8Br-cGMP) on I_Na_. (**A**, **B**) Representative I_Na_ traces with vehicle and 500 μM 8Br-cGMP applied for 24 h, and their I–V relationships of I_Na_. 8Br-cGMP was unable to modify I_Na_. Numbers of cell are shown in parentheses. Values represent the mean ± SE.

The existence of two effector pathways of NO, NO/cGMP/PKG signal cascade and NO/S-nitrosylation dependent signal pathway, has important functional implications on cardiovascular system. To clarify the mechanism of NOC-18 for I_Na_ modulation, we have applied NOC-18 in the presence of a protein kinase G inhibitor KT5823 (Fig. 5A,B). The reduction of I_Na_ by NOC-18 occurred even when cardiomyocytes were incubated with KT5823. Importantly, reduction rate of I_Na_ by NOC-18 in the presence of KT5823 (29%) was nearly identical to those without KT5823 (26%) in Fig. 1, indicating that effect of NO on I_Na_ suppression is independently of the signal pathway caused by cGMP/PKG. This observation was also confirmed by the experiment by use of a guanylate cyclase inhibitor 1H-^1,2,4^ oxadiazolo[4,3,-a]quinoxalin-1-one (ODQ); reduction of I_Na_ by NOC-18 was unmasked by ODQ (Fig. 5C,D). Furthermore, a membrane permeable cGMP analogue, 8-Bromo-cGMP (8Br-cGMP), had no effect on I_Na_ (Fig. 6), which firmly supports the conclusion that GC/cGMP/PKG signaling pathway is not involved in NOC-18-induced long-term inhibition of I_Na_.

### Possible interaction with a transcriptional regulator FOXO1

**Figure 7:**
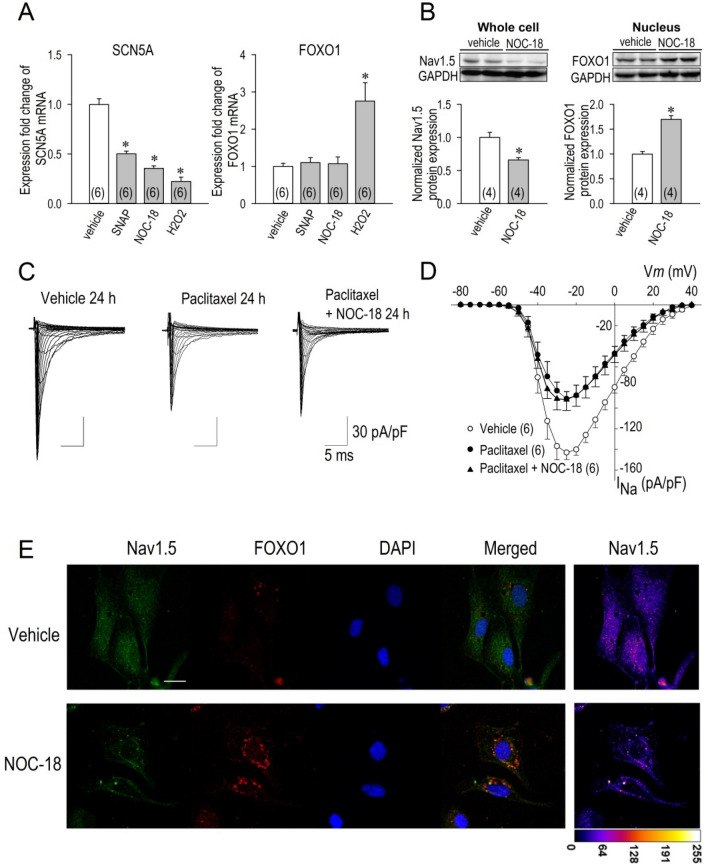
FOXO1 as a possible link between NO and Nav1.5. (**A**) Changes of SCN5A mRNA- and FOXO1 mRNA-expression were examined by NO donors SNAP and NOC-18. SNAP (500 μM) and NOC-18 (1 mM) significantly reduced mRNA levels of SCN5A, although both NO donors were without effects on FOXO1 mRNA levels. Effects of 25 μM H_2_O_2_ were assessed as positive controls. (**B**) Effects of NOC-18 on SCN5A and FOXO1 protein expression evaluated by Western blot analysis. Nav1.5 protein levels (whole cell) were significantly reduced, whereas FOXO1 protein levels (nucleus) were significantly increased by 1 mM NOC-18 applied for 24 h. Numbers of sample are shown in parentheses. Data were expressed as mean ± SD. *p < 0.05. See the supplemental file for the complete gel/blot images. (**C**,**D**) Actions of a FOXO1 activator paclitaxel and NOC18 for I_Na_. (**C**) Representative I_Na_ traces with vehicle, 1 μM paclitaxel and 1 μM paclitaxel with 1 mM NOC-18 applied for 24 h, and (**D**) their I–V relationships. Numbers of cell are shown in parentheses. (**E**) Expression and distribution of Nav1.5 and FOXO1 as assessed by immunocytochemistry procedure. Cardiomyocytes were exposed to vehicle or 1 mM NOC-18 for 24 h. Nav1.5 for stained in green, FOXO1 in red, and DAPI staining to visualize nuclei in blue. Scale bar = 20 μm.

NO modulation of I_Na_ may be caused by inhibition of the channel synthesis, which includes regulation of mRNA transcription and protein expression. Therefore we did real-time RT-PCR quantification of the cardiac Na^+^ channel genes, SCN5A, using mRNA samples extracted from cardiomyocytes treated with or without NO donors for 24 h. As shown in Fig. 7A, the mRNA levels were significantly reduced by NO donors, SNAP and NOC-18. Hydrogen peroxide (H_2_O_2_) is a positive control which is known to reduce SCN5A and increase a transcription factor forkhead box protein O1 (FOXO1) in cardiomyocytes^21^. In contrast to SCN5A, FOXO1 mRNA was unchanged by SNAP or NOC-18 (Fig. 7A). We then carried out Western blot analysis of the Na^+^ channel and FOXO1 proteins isolated from neonatal rat cardiomyocytes. Figure 7B demonstrates that 1 mM NOC-18 reduced Nav1.5 protein expression by 34% (n = 4), and increased FOXO1 protein by 70% (n = 4) in the nucleus. In consistent with this result, a FOXO1 activator paclitaxel, applied for 24 h, significantly decreased I_Na_: the maximum inward current of I_Na_ was reduced by 36% (Fig. 7C,D). When NOC-18 was applied with paclitaxel, I_Na_ was reduced by 36%, which was identical to those of paclitaxel (Fig. 7C,D). We note that the reduction ratio of the maximum inward current of I_Na_ by 1 mM NOC-18 alone was 26% (Fig. 1C). Taken together with this reduction ratio, it is suggested that NOC-18 is without effect on I_Na_ when FOXO1 is highly activated. Furthermore, immunocytochemistry staining revealed that NOC-18 exhibited disarrangement and suppression of Nav1.5 protein accompanied by a perinuclear aggregation of FOXO1 protein in cardiomyocytes (Fig. 7E). Thus, these results lead to the conclusion that decreased Nav1.5 protein levels by NOC-18 may be due to the modulation of FOXO1 activity as a repressor to inhibit the transcription of SCN5A in cardiomyocytes.

## Discussion

The present study demonstrates a novel NO-dependent modulatory mechanism that alters the voltage-gated Na^+^ channel protein Nav1.5 and thereby I_Na_ in neonate cardiomyocytes. Although NO is recognized as a potent vasodilator, and its activity is highly beneficial for the ischemic heart, this investigation provide a surprising new dimension to NO signaling which is the cGMP-independent action of NO to down-regulate the Na^+^ channel through S-nitrosylation. The major findings of this investigation are summarized as follows: (1) Long-term but not short-term application of NO donors, NOC-18 and SNAP, reduced I_Na_ in a dose-dependent manner; (2) an NO donor NOC-18 shifted the steady-state inactivation curve, but not the activation curve, to the hyperpolarization direction; (3) a potent NO inhibitor Carboxy-PTIO masks the effect of NOC-18 on I_Na_; (4) a GC inhibitor ODG and a PKG inhibitor KT5823 could not block the effect of NOC-18 on I_Na_, (5) a membrane permeable cGMP (8 Br-cGMP) was without effect on I_Na_; (6) protein thiol modulation inhibitors, NEM and DTE, suppressed the effect of NOC-18 on I_Na_, (7) NOC-18 decreased the protein expression of Nav1.5 channel, and increased the expression of a transcription factor FOXO1 in the nucleus, (8) NOC-18 was without effect on I_Na_ when FOXO1 was highly activated by a FOXO1 activator paclitaxel, and (9) immunocytochemistry staining revealed that NOC-18 suppressed Nav1.5 protein expression accompanied by a perinuclear aggregation of FOXO1 protein. These findings suggest a novel action of NO on the voltage-gated Na^+^ channel through S-nitrosylation pathway, which could be adverse to cardiac function particularly in the diseased conditions of the heart.

NO is produced from virtually all cell types composing the myocardium, and regulates cardiac function through vascular-dependent and—independent manners. The role of NO in cardiac function is complex and controversial^22^. NO can bind to GC, increasing cGMP production and activate PKG. NO may also directly S-nitrosylate cysteine residues of specific proteins. In this context, it is particularly important to elucidate molecular targets of NO, or NO donors NOC-18 and SNAP in this experiment. For voltage-gated ion channels, targets of PKG include Ca^2+^-activated K^+^ channel^23^, ATP-sensitive K^+^ channel^24^, voltage-gated L, N, T-type Ca^2+^ channel^25,26^, and voltage-gated Na^+^ channels^27^ with their respective impact on cellular excitability. In addition, S-nitrosylation of cysteine residues has emerged as an important feature of NO signaling. Though this post-translational modification, NO is able to regulate the function of ion channels including, Ca^2+^ activated K^+^ channel^28^, voltage-gated L-type Ca^2+^ channel^29^, cyclic nucleotide gated channel^30^, and voltage-gated Na^+^ channels^4,31^. In addition to these emerging evidence linking PKG/S-nitrosylation to the voltage-gated Na^+^ channel by regulating the state of post-translational modulation, this investigation, for the first time, shed light on the transcriptional effects of NO on the cardiac Na^+^ channel.

In the present study NOC-18 and SNAP were used as NO donors. It has been reported that NO released from 1 mM NOC-18 results in steady-state levels of 1–5 μM NO in medium without any cofactors^32^. This is comparable to concentrations (1–30 μM) produced by endogenous inducible NO synthase in culture media and in plasma after cytokine stimulation or lung injury^33^. For the clinical setting of NO for the treatment of pulmonary hypertension, acute lung injury, and cardiopulmonary failure, concentration of 15–30 ppm (5–10 μM) of NO has widely been applied^34^. During these clinical applications of NO, lung endothelium and cardiomyocytes are exposed to be exposed to the same concentration or slightly lower than that in the inhaled NO gas. Thus exposure of cardiomyocytes to 1–5 μM NO constitutes a pathophysiologically relevant cellular model with which to study NO-mediated modulation of ion channels in cardiomyocytes. Cultured neonatal cardiomyocytes were exposed to 1 mM NOC-18 for 24 h in this investigation. Therefore results in this study imply that NO plays an important role in regulation of myocardial Na^+^ channel during clinical therapeutic application of NO or NO donor in vivo.

Although our study could not detect the acute effects of NO donor on I_Na_, previous several studies identified NO/cGMP/PKG pathway to regulate I_Na_ as a short-term effect^16,27,35^. Because the life span of NO in blood milieu or in the bathing solution is less than 0.2 ms^30^, biological exposure time of NO largely depends on the NO-releasing speed of NO donor. NOC-18 is a diazeniumdiolate slow-releasing NO donor with a half-life for NO release of 20 h, where the rate of release is attributed to the structure^36^. NOC-18 is an ideal NO donor since the amine byproduct formed has no known interferences with cellular activities. Therefore, unlike experiments using gaseous NO to obtain saturated concentration of NO in the medium, experiments by use of a slow-releasing NO donor NOC-18 are suitable to assess the possible transcriptional effect of NO to cardiomyocytes. In this context, we could not observe persistent Na^+^ current in this experiments, presumably because increases of NO concentration by slow-releasing NO donors were neither prompt and high enough in the bath medium for the electrophysiological studies. Also as a limitation of whole-cell patch clamp experiments, in most patch clamp amplifier in the market, leak subtraction could subtract the leak current produced by single depolarizing pulse but not the leak current produced by a series of different step depolarizing pulses. Due to its assumption that leak current would be produced if only potential difference arises across membrane, recording protocols in manufactured voltage-clamp program by use of leak subtraction are not suitable to subtracting the steady-state leak current while recording voltage-gated channel currents which is also time-dependently altered.

Several studies, particularly based on plants with altered NO levels, have recently provided genetic evidence for the importance of NO in gene induction^37,38^, although little is known on the role of NO as a regulator of gene expression in mammals. Furthermore, the NO-dependent intracellular signaling pathway(s) that lead to the activation or suppression of these genes have not yet been defined. In this context, for the first time, our study has revealed a novel role of NO as a positive modulator of FOXO1 in cardiomyocytes, leading to a reduction of Nav1.5 proteins. Because FOXO1-mRNA was unchanged and phosphorylated form of FOXO1 was increased in the nucleus in response to NO (Fig. 7A,B), and because NOC-18 suppressed Nav1.5 protein expression accompanied by a perinuclear aggregation of FOXO1 protein (Fig. 7E), it is suggested that FOXO1 translocates from the cytoplasm to the nucleus and suppresses the expression of Nav1.5. Phosphorylated Foxo1 can be dephoshosphorylated by phosphatase allowing Foxo1 to enter the nucleus^39^. Therefore functional modulation or stimulation of phosphatase by NO-mediated S-nitrosylation is possibly postulated, although we have no data to support this speculation. Obviously a complete transcriptome analysis is needed for the understanding about the mechanism of NO-mediated Nav1.5 suppression.

Derangement of NO production regulation, such as produced on excessive NO delivery from inflammatory cells (or cytokine-stimulated cardiomyocytes themselves), may result in profound cellular disturbances leading to heart failure^40^. At the same time, however, the functional consequences of altered NO synthase expression and NO bioavailability in the failing heart are poorly characterized. Namely, quite a few numbers of diverse and often contradictory effects of NO and NO donors on myocardial function have been reported. It is widely accepted that NO could modulate inotropic, chronotropic, and dromotropic response to β adrenoceptor stimulation: low dose enhances and high dose reduce β adrenergic response^41^. Interestingly, an action of NO to modulate β adrenergic inotropic responses in humans in vivo could only be demonstrated in patients with heart failure and not in “normal” subjects^42^. In relation to the action of NO to heart failure, we note the possible adverse effect of NO in patients with diseased heart. In human study, prolonged nitrate treatment was reportedly not beneficial for patients with myocardial infarction^,43,44^ and heart failure^45^.

**Figure 8:**
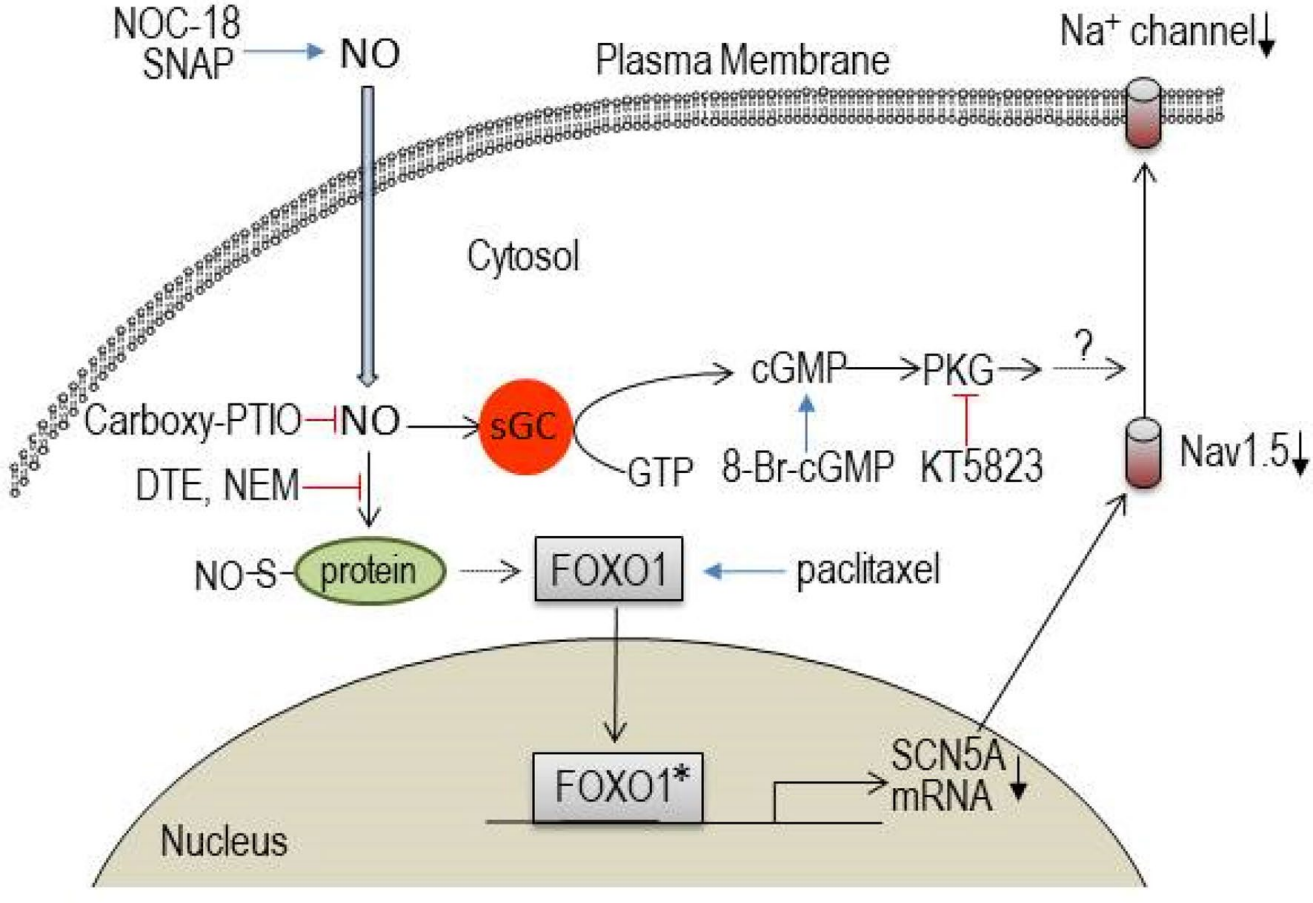
The current hypothesis regarding NO-mediated downregulation of the Na^+^ channel. NO is suggested to act through a suppressor gene FOXO1 for SCN5A transcription. FOXO1*: activated form of FOXO1.

Although we have successfully demonstrated that NO down-regulates I_Na_ in neonatal cardiomyocytes, and have postulated a possible FOXO1-dependent signaling pathway for the regulation of Nav1.5 (Fig. 8), we still need to identify interactor molecules between NO and FOXO1 transcription. As limitation of this investigation, it is also important to keep in mind that reduction of I_Na_ and the sodium channel protein was confirmed by NO donors in *in-vitro* experiments but not in *in-vivo* condition of the heart. Because physiological actions of NO in the heart are largely dependent on the vascular and neuronal regulation of the circulation, drug actions on cardiomyocytes without systemic circulation may not accurately represent the clinical pharmacological effects of NO. Furthermore, we are not sure whether NO donors mimic endogenous NO-related response in the heart. In addition, we could not explore the action of NO on the accessory proteins of the channel including β1 and β2 subunits, which may affect the gating properties of the channel; e.g. the shift of the steady-state inactivation curve to the hyperpolarization direction. Furthermore, this study is largely dependent upon electrophysiological evaluation of I_Na_. Therefore, molecular biological analyses for S-nitrosylation substitutes or interactor molecules, quantitative FOXO1 activity, potentially interacted transcription factors besides FOXO1, and mechanisms of FOXO1 nuclear translocation were not performed. Thus, obviously future studies are needed to address these questions.

We conclude that NO is a negative modulator of the voltage-gated Na^+^ channel in cardiomyocytes. A significant reduction of I_Na_ as well as the SCN5A protein is considered to be one of mechanisms possibly related to NO-induced cardiac dysfunction particularly in heart failure. The endogenous mechanisms of transcriptional regulation of SCN5A in cardiomyocytes are largely unknown. We also demonstrate in this study that increase of a transcription factor FOXO1 in the nucleus is the trigger for the down-regulation of SCN5A in NO-treated cardiomyocytes. These findings indicate that many diverse and often contradictory effects of NO or NO donor on myocardial function could be attributed to the conduction defect and/or arrhythmias in the heart, at least in a part, caused by a reduction of the voltage-gated Na^+^ channel.

## Methods

### Neonatal rat cardiomyocytes: preparations and culture

All experimental protocols were approved in advance by the Ethics Review Committee for Animal Experimentation of Oita University School of Medicine (No. 1704001, No. 1704002), and were carried out according to the guidelines for animal research of the Physiological Society of Japan to minimize the number of animals used as well as their suffering. Neonatal cardiomyocytes were prepared from 1- to 3-day old Wistar rats as described previously^46^. The cardiomyocytes were plated onto 35-mm culture dishes and cultured in Dulbecco’s Modified Eagle’s Medium, supplemented with 5% fetal bovine serum at 37 °C under 5% CO_2_. The cells were seeded onto glass-bottom dishes and incubated in a culture medium for 24–48 h before electrophysiological measurements.

### Electrophysiological measurements

Macroscopic Na^+^ channel currents were recorded in whole cell configuration using an EPC-9 amplifiers (HEKA Electronik, Lambrecht, Germany) at room temperature (20–23 °C) as described previously^47^. Patch pipettes were pulled from 75-mm plain capillary tubes (Drummond Scientific Co., Broomall, PA, USA) with a micropipette puller, Model P-97 (Sutter Instrument, Co., Novato, CA, USA), and were fire-polished subsequently. The electrode had a resistance of 1.5–2.5 MΩ when the pipette was filled with the pipette solution (see below). Series resistance was compensated electrically as much as possible without oscillation (60–75%). Capacitive artifacts were minimized by using the built-in circuitry of the amplifier. The current signals were filtered at 3.3 kHz and digitized at 10 kHz under the control of a data acquisition program, Pulse/Pulsefit (V.8.11, HEKA Electronik). To investigate the channel availability (steady-state inactivation), a conventional double-pulse protocol was applied every 2 s: 50 ms of test pulses at − 10 mV following 200 ms of prepulses from − 140 to − 10 mV (increment = 5 mV) were applied. The reversal potential and the chord conductance were calculated by fitting the current–voltage (I–V) relationship to a Boltzmann distribution function: *I* = G_max_ (V_m_ − V_rev_ )/(1 + exp[(V_m_ − V_a, 1/2_ )/k]), where *I* is the peak *I*_Na_ at the given test potential V_m_ , V_rev_ is the reversal potential, G_max_ is the maximal chord conductance, V_a, 1/2_ is the half-point of the relationship and k is the slope factor. The voltage-dependent inactivation was similarly determined with a Boltzmann equation: *I*/*I*_max_ = 1/(1 + exp[(V_m_ − V_i, 1/2_ )/k]), where V_m_ is the membrane potential, V_i, 1/2_ is the half-point of the relationship and k is the slope factor. The recording chamber was filled with the bath solution of the following components (mM): NaCl 30, MgCl_2_ 0.5, TEA-Cl 125, CsCl 5, 4-AP 5, DIDS 0.1, HEPES 10, glucose 10, CaCl_2_ 1.8 (pH of 7.4 adjusted with 1* N* TEA-OH). The patch-clamp electrode was filled with the pipette solution of (mM): CsF 20, CsCl 120, EGTA 2, HEPES 5, (pH of 7.2 adjusted with 1 N CsOH). The data were acquired by using computer software (Pulse/Pulsefit, V.8.11), and all curve fittings were made on SigmaPlot (V10.0, SPSS Inc., Chicago, IL, USA).

### Quantitative real-time RT-PCR

Total RNA was extracted from rat neonatal cardiomyocytes by using TRIzol (Invitrogen, Carlsbad, CA) 24 h after the treatment with agents as described previously^48^. The cDNA was synthesized from 1 mg of total RNA by using Transcriptor First Strand cDNA Synthesis Kit (Roche Molecular System Inc., Alameda, CA, USA). The real-time PCR was performed on Light Cycler (Roche) by using the FastStart DNA Master SYBR Green I (Roche) as a detection reagent. The sequences of the specific primers were; (SCN5A; KM373692) forward 5′-caggtcggaaacttggtcttcac-3′ and reverse 5′-ggaacgcagcacagaca-3′; (Foxo1; NM_019739) forward 5′-ctcccggtacttctctgctg-3′ and reverse 5′-gtggtcgagttggactggtt-3′; (GAPDH; GU214026) forward 5′-ccacccagaagactgtggat-3′ and reverse 5′-cacattgggggtaggaacac-3′. Data were calculated by 2^–ΔΔCT^ and presented as fold change in transcripts for SCN5A and Foxo1 genes and normalized to GAPDH (defined as 1.0-fold).

### Western blot analysis

Western blot analysis was performed as described previously^49^. Cells were rinsed with phosphate-buffered saline (PBS) twice, and protein was extracted in cold whole cell lysis buffer (Cell Signaling, Beverly, MA). For preparation of nuclear extracts, cells were washed twice with ice-cold PBS, scrapped, and transferred to a centrifuge tube. Nuclear protein from cardiomyocytes were prepared using NE-PER Nuclear and Cytoplasmic Extraction Reagents based on the manufacturer’s instruction (Pierce, Rockford, IL). The extracted protein concentration was determined by the BCA protein assay kit (Pierce, Rockford, IL). Forty μg of protein samples was loaded on equal protein basis, separated on a 10% SDS-PAGE and transferred from the gel to a PVDF membrane (Hybond-P; GE Healthcare Bio-Sciences, Piscataway, NJ, USA). The membrane was blocked by using 5% skim milk in Tris-buffered saline containing 0.05% Tween 20 for 1 h and then incubated with the indicated primary antibody overnight at 4 °C. The primary antibodies used were as follows: rabbit anti-Foxo1 polyclonal (Cell Signaling, 1∶1000), rabbit anti-Na_V_1.5 polyclonal (Alomone Labs Ltd., Jerusalem, Israel, 1∶200). The blot was visualized with anti-rabbit IgG horseradish peroxidase-conjugated secondary antibody (1:2000, American Qualex, CA) and an ECL plus Western Blotting Detection System (GE Healthcare Bio-Sciences) by using the Image Quant LAS 4000 mini (GE Healthcare GE Healthcare Bio-Sciences) imaging system. Densitometric analysis of each band was carried out using ImageJ software (Wayne Rasband, National Institutes of Health). Protein loading was controlled by probing Western blots with anti-β-actin (1:1000, Cell Signaling) and normalizing Na_V_1.5 and Foxo1 protein band intensities to β-actin values.

### Immunocytochemistry

The details of the experiments protocol were performed as described previously^50^. Primary antibodies against Foxo1 (1:200, Cell Signaling, Beverly, MA, USA) and Nav1.5 (1:200, Alomone Labs Ltd., Jerusalem, Israel) were applied following by incubation with the appropriate fluorescence-labeled secondary antibodies (Invitrogen) for 1 h at room temperature. After several washes, the samples were air-dried, mounted with a drop of ProLong Diamond Antifade Mountant with DAPI (Molecular Probes) and subjected to microscopy. Images were obtained with a confocal microscope system (A1R, Nikon, Tokyo, Japan) equipped with a PlanFluor 60X objective lens and excitation lasers (488 and 561 nm, Melles Griot). Images were saved in TIFF format and analyzed by ImageJ software (Wayne Rasband, National Institutes of Health).

### Data analysis

IC_50_ values were estimated using least squares linear regression programmed in SigmaPlot (V10.0, SPSS Inc.). The group data show means ± SE or SD. Between groups and among groups comparisons were conducted and evaluated by a Tukey–Kramer test, and between two groups, a Brunner-Munzel test was used. *p* < 0.05 was considered statistically significant.

### Drugs

All other chemicals were purchased from Wako Chemical Co., Osaka, Japan.

## Supplementary Information

Supplementary Information.

## Data Availability

The datasets generated during and/or analyzed during the current study are available from the corresponding author upon reasonable request.
