# Supplementary material for: Nitric oxide down-regulates voltage-gated Na+ channel in cardiomyocytes possibly through S-nitrosylation-mediated signaling
Source: Sci Rep. 2021 May 28;11:11273. doi: 10.1038/s41598-021-90840-0 (PMC8163867; doi:10.1038/s41598-021-90840-0)
Supplement: Supplementary file 1 — Supplementary Information. [file 41598_2021_90840_MOESM1_ESM.pdf]

Nitric oxide down-regulates voltage-gated Na^+ channel in cardiomyocytes possibly through S-nitrosylation-mediated signaling

Pu Wang[1,2], Mengyan Wei[1,2], Xiufang Zhu[1,2], Yangong Liu[1,2], Kenshi Yoshimura[2], Mingqi Zheng[1], Gang Liu[1], Shinichiro Kume[2], Masaki Morishima[3], Tatsuki Kurokawa[2], Katsushige Ono[2]
[1]Department of Cardiology, The First Hospital of Hebei Medical University, 89 Donggang Road, Shijiazhuang, Hebei Province 050031, People's Republic of China
[2]Department of Pathophysiology, Oita University School of Medicine, Yufu, Oita 879-5593, Japan
[3]Department of Food Science and Nutrition, Faculty of Agriculture, Kindai University, Nara, Japan
Correspondence: Katsushige Ono, ono@oita-u.ac.jp

Supplemental Data

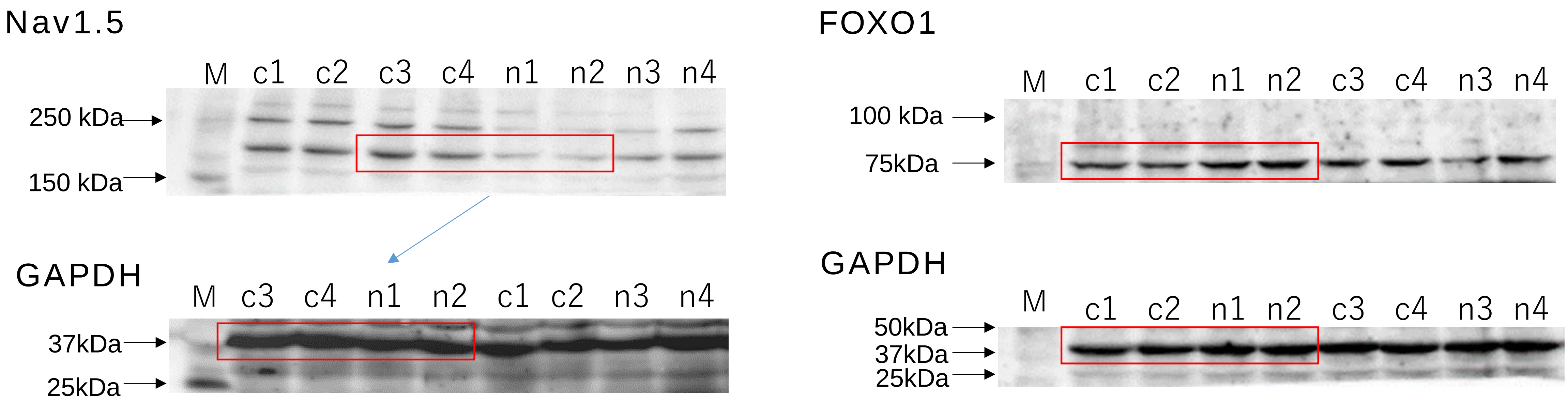

Western blot in Figure 7 corresponds to this slide data indicated by red box. Blot labels c and n represent vehicle (c) and NOC-18 (n), respectively. Blot data c3 and c4 were used for vehicle and n1 and n2 were used for NOC-18 in Figure7B.
